# Supplementary material for: Safety, tolerability, pharmacokinetic and pharmacodynamic effects of the muscarinic M1 positive allosteric modulator VU0467319 for Alzheimer’s disease: a single ascending-dose study in healthy participants
Source: Alzheimers Res Ther. 2025 Jul 1;17:144. doi: 10.1186/s13195-025-01798-4 (PMC12211128; doi:10.1186/s13195-025-01798-4)
Supplement: Supplementary file 1 — Supplementary Material 1 [file 13195_2025_1798_MOESM1_ESM.docx]

Supplementary Materials

Safety, Tolerability, Pharmacokinetic and Pharmacodynamic Effects of the Muscarinic M1 Positive Allosteric Modulator VU0467319 for Alzheimer’s disease: A Single Ascending-Dose Study in Healthy Participants

### Appendix 1. Description of the Exploratory Pharmacodynamics: Cognitive and Electrophysiological Measures

Appendix 1.1: Cognitive Attention Tasks

Critical Flicker Fusion Task

The critical flicker fusion task (CFF; (Kupke & Lewis, 1989)) was used to test perceptual vigilance using the frequency of a flickering LED (between 12-50 Hz). The outcome variable for CFF is frequency (Hz) for ascending and descending trials. In the ascending trials, the participants pressed a button indicating when the light flickered to a speed where they appear to be continuously on or fused. In the descending trials, the participants pressed a button when the frequency of apparently fused lights is decreased such that lights begin to appear to be flashing.

Choice Reaction Time Task

The choice reaction time (CRT; (Hindmarch, 1984)) task was used to measure psychomotor speed. Outcome variables on the CRT were the median total reaction time, which can be broken down into the recognition time (time from stimulus onset to initiation of movement), and the motor reaction time (time from initiation of movement to stimulus termination).

Conner’s Continuous Performance Task-II

The Conner’s Continuous Performance Task (CPT; (Conners et al., 2000)) tested sustained attention to a target. Participants saw a string of letters appearing one at a time on a computer screen. Participants were asked to press the spacebar whenever the target stimulus (any letter except for the X) appeared. Participants were asked to withhold responding when a X was presented, which is the non-target stimulus. A set of 15 outcome measures derived from the task report were recorded as key variables. These measures include Hit RT, Hit Standard Error, Commission, Omission and Perseveration errors, Sensitivity, Response style, and the Hit standard error across different interstimulus intervals.

Spatial Selective Attention Test

A version of the spatial selective attention test (SSAT; (Posner, 1980)) was used to assess the ability of participants to disengage attention and shift to a new target. Participants were asked to press a button corresponding to the side of the screen on which a stimulus appeared. A central diamond was placed in between serving as a fixation point. Cues consisted of a 200-ms darkening of one side of the diamond depicting an arrowhead pointing to one of the peripheral boxes. The cue was followed by a target appearing for 100-ms in one of the boxes. To prevent temporal orienting, we used two randomly occurring cue–target intervals (400 and 700-ms). The targets were black diamonds that appeared in either the left or right boxes. In most trials, the cue validly predicted the target, however some cues were invalid, that is they pointed in the opposite direction of the target. On some trials, both sides of the diamond darkened, resulting in an ambiguous or neutral cue in which the participants were not able to prepare a specific hand to respond. On other trials, the cue did not show at all before the presentation of the target, giving participants no time to prepare a response. The task consisted of 112 trials, with 64 trials (57%) with valid cues, and 16 trials (14.3%) each for the invalid, neutral or no cues conditions. The outcome variables of the task are the reaction time effects related to the ability of participants to anticipate a target when there is an ambiguous cue compared to there being no cue at all (Alerting effect: no cue – neutral cue); and the ability of participants to shift their attention between congruent and incongruent stimuli (Reorienting effect: invalid cue – valid cue).

Appendix 1.2: Cognitive Memory Tasks

Verbal N-back Task

A visually presented N-back sequential letter task (Jonides et al., 1997; Saykin et al., 2004) was used to assess working memory performance, wherein participants saw a string of consonants (except L, W, and Y), presented in upper case letters, one every 3 s. Four conditions were presented: 0-back, 1-back, 2-back, and 3-back. The 0-back control condition required participants to decide if the current letter matched a single target letter that was specified before the epoch began. In each of the 1-back, 2-back, and 3-back conditions, the task is to decide whether the letter currently presented matches the letter that has been presented 1, 2, or 3 back in the sequence. The 0-, 1-, 2-, and 3-back conditions were performed in two blocks of 27 trials each for a total of 216 trials. The blocks were presented in a counterbalanced order such that the same condition was not repeated two times in a row. The main outcome variable of the n-back task was sensitivity (*d’*) for each of the four conditions, calculated as: *d’* = Z (Hit) – Z (False alarms).

Selective Reminding Task

The Selective Reminding Task (SRT; (Buschke & Fuld, 1974)) was used to test immediate and delayed episodic recall. Participants were read a list of 16 words and must immediately recall the list across 8 trials. Following each trial, participants were reminded of the words that they did not recall. Upon completing the immediate recall portion of the SRT and after a 20‐minute delay, participants were asked to complete a single delayed recall trial. SRT total immediate recall was analyzed using the number of correctly recalled words across trials 1–8, total immediate recall consistency was analyzed using the number of words correctly recalled on two sequential trials across trials 1–8, SRT total immediate recall failure was analyzed using the number of words not recalled on two sequential trials across trials 1–8, and total delayed recall was analyzed using the number of words correctly recalled after a 20-min delay.

Appendix 1.3: Electrophysiological Tasks

Oddball Tasks

Auditory and visual oddball tasks were performed to assess attention in auditory and visual modalities (Comerchero & Polich, 1999; Polich, 2007). In the auditory task, a pair of two pure tones (single formant) at 1000 and 1500 Hz were the stimuli. Tones were equated in duration (300 ms) and rise/decay times. Tones were presented at 75 dB SPL (measured at the ear) through a speaker positioned 1 meter in front of the participant. In the visual task, the stimuli were a pair of letters (X & O). All stimuli were presented in random order for 500 ms in the center of a computer monitor positioned 1 meter in front of the participant. For both auditory and visual tasks, 200 trials were presented with the inter-stimulus interval varying randomly between 1000-1300 ms to prevent habituation to stimulus onset. The assignment of stimuli to standard and target conditions (70% & 30% of the trials respectively), was counterbalanced across participants. Each participant was asked to press a different button using their preferred hand upon presentation of the standard and the target stimulus. On average, auditory and visual tasks lasted 6-7 minutes each.

Incidental Memory Task

A passive incidental visual memory paradigm (120 trials, 8 min; (Key & Dykens, 2014)) was used to evaluate changes in basic memory processes. Participants were asked to view a slide show of novel color photographs depicting complex natural scenes (different sets of images were used at pre-dose vs. post-dose administration test sessions). A small subset (*n* = 10) of the stimuli was presented repeatedly (× 5 each) throughout the session; the rest (*n* = 50) were shown once. All stimuli were presented in random order for 1500 ms with a random interstimulus interval of 1300-1600 ms. To encourage attention to the stimulus sequence, 20 attention probes (a yellow smiley face) were presented throughout the test session and required a button press response. The task duration was approximately 7 minutes.

Appendix 1.4 References

Buschke, H., & Fuld, P. A. (1974). Evaluating storage,retention, and retrieval in disordered memory and learning. *Neurology*, *24*(November), 1019–1025.

Comerchero, M. D., & Polich, J. (1999). *P3a and P3b from typical auditory and visual stimuli*. *110*, 24–30.

Conners, C. K., Staff, M. H. S., Connelly, V., Campbell, S., MacLean, M., & Barnes, J. (2000). Conners’ Continuous Performance Test II (CPT II V. 5). *Multi-Health Systems Inc*, *29*, 175–196. https://doi.org/10.1207/s15326942dn2901_9

Hindmarch, I. (1984). Psychological performance models as indicators of the effects of hypnotic drugs on sleep. *Psychopharmacology. Supplementum*, *1*, 58–68.

Jonides, J., Schumacher, E. H., Smith, E. E., Lauber, E. J., Awh, E., Minoshima, S., & Koeppe, R. A. (1997). Verbal working memory load affects regional brain activation as measured by PET. *Journal of Cognitive Neuroscience*, *9*(4), 462–475. https://doi.org/10.1162/jocn.1997.9.4.462

Key, A. P., & Dykens, E. M. (2014). Event-related potential index of age-related differences in memory processes in adults with down syndrome. *Neurobiology of Aging*, *35*(1), 247–253. https://doi.org/10.1016/j.neurobiolaging.2013.07.024

Kupke, T., & Lewis, R. (1989). Relative influence of subject variables and neurological parameters on neuropsychological performance of adult seizure patients. *Archives of Clinical Neuropsychology*, *4*(4), 351–363. https://doi.org/10.1016/0887-6177(89)90025-5

Polich, J. (2007). Updating P300: An integrative theory of P3a and P3b. *Clinical Neurophysiology*, *118*(10), 2128–2148. https://doi.org/10.1016/j.clinph.2007.04.019

Posner, M. I. (1980). Orienting of attention. *Quarterly Journal of Experimental Psychology*, *32*(1), 3–25. https://doi.org/10.1080/00335558008248231

Saykin, A. J., Wishart, H. A., Rabin, L. A., Flashman, L. A., McHugh, T. L., Mamourian, A. C., & Santulli, R. B. (2004). Cholinergic enhancement of frontal lobe activity in mild cognitive impairment. *Brain*, *127*, 1574–1583. https://doi.org/10.1093/brain/awh177

### Appendix 2. Supplementary Tables

See below detailed tables for urine PK concentrations of VU319, and both plasma and urine PK concentrations of VU424; as well as an expanded table of TEAEs; and also results of the exploratory cognitive and electrophysiological tasks across both the single ascending dose and food effect studies.

Supplementary Table 1. Plasma Pharmacokinetic Parameters of VU424 Across Single Ascending Dose Cohorts and Food Conditions

|  | Single Ascending Dose Cohorts | | | | | | | | | | Food Effect Study | | | |
| --- | --- | --- | --- | --- | --- | --- | --- | --- | --- | --- | --- | --- | --- | --- |
| VU319 Dose | **60 mg** | | **120 mg** | | **240 mg** | | **400 mg** | | **600 mg** | | **120 mg Fasted Condition** | | **120 mg Fed Condition** | |
| PK Parameter | **Mean (SD)** | **N** | **Mean (SD)** | **N** | **Mean (SD)** | **N** | **Mean (SD)** | **N** | **Mean (SD)** | **N** | **Mean (SD)** | **N** | **Mean (SD)** | **N** |
| C_max_ (μg/mL) | 0.231 (0.076) | 6 | 0.29 (0.098) | 6 | 0.45 (0.18) | 6 | 1.26 (0.57) | 6 | 3.78 (0.48) | 6 | 0.38 (0.16) | 10 | 0.47 (0.158) | 10 |
| T_max_ (h)^a^ | 24.0 (7.02, 48.0) | 6 | 41.9 (12.0, 48.4) | 6 | 36.0 (24.0, 48.4) | 6 | 42.0 (5.0, 48.2) | 6 | 47.9 (5.0, 97.0) | 6 | 48.0 (24.0, 48.1) | 10 | 24.0 (9.0, 48.1) | 10 |
| AUC_0-last_ (μg•h/mL) | 20.6 (7.13) | 6 | 27.2 (7.51) | 6 | 33.0 (19.6) | 6 | 120 (58.3) | 6 | 344 (77.5) | 6 | 32.6 (12.4) | 10 | 34.8 (6.73) | 10 |
| AUC_0-∞_ (μg•h/mL) | 20.6 (7.86) | 4 | 34.0 (11.1) | 2 | 58.8 (N/A) | 1 | 122 (56.8) | 2 | 337 (64.7) | 4 | 35.9 (15.1) | 8 | 38.7 (9.16) | 8 |
| %AUC_ext_ (%) | 6.44 (2.58) | 4 | 9.01 (7.61) | 2 | 2.06 (N/A) | 1 | 10.6 (5.53) | 2 | 10.5 (4.89) | 4 | 8.48 (4.42) | 8 | 8.26 (5.49) | 8 |
| t_½_ (h) | 36.1 (6.47) | 4 | 41.1 (16.6) | 2 | 25.1 (N/A) | 1 | 43.4 (10.6) | 2 | 40.3 (11.8) | 4 | 43.6 (22.0) | 10 | 45.0 (27.2) | 10 |
| C_max_/Dose (ng/mL/mg) | 3.86 (1.26) | 6 | 2.45 (0.82) | 6 | 1.85 (0.76) | 6 | 3.14 (1.43) | 6 | 6.31 (0.79) | 6 | 3.17 (1.33) | 10 | 3.95 (1.31) | 10 |
| AUC_0-last_/Dose (ng•h/mL/mg) | 335 (119) | 6 | 226 (62.6) | 6 | 138 (81.8) | 6 | 301 (146) | 6 | 574 (129) | 6 | 271.3 (102.8) | 10 | 289.5 (56.03) | 10 |
| AUC_0-∞_/Dose (ng•h/mL/mg) | 343 (131) | 4 | 284 (92.4) | 2 | 245 (N/A) | 1 | 306 (142) | 2 | 562 (108) | 4 | 299.4 (125.66) | 8 | 322.75 (76.45) | 8 |

Notes. %AUC_ext_=percentage of area under the curve extrapolated from time T_last_ to infinity; AUC_0-last_=area under the plasma concentration-time curves from time 0 to the time of the last detectable concentration; AUC_0-last_/Dose=AUC_0-last_ normalized to dose; AUC_0-∞_=area under the plasma concentration-time curve from time 0 to infinity; AUC_0-∞_/Dose=AUC_0-∞_ normalized to dose; C_max_=maximum observed plasma concentration; C_max_/Dose=C_max_ normalized to dose; N=number of subjects; N/A=not available; PK=pharmacokinetic; SD=standard deviation; t_½_=apparent terminal elimination half-life; T_max_=time to maximum plasma concentration; T_last_=time of last detectable concentration. ^a^T_max_ values are presented as median (minimum, maximum).

Supplementary Table 2. Urine Pharmacokinetic Parameters of VU319 Across Single Ascending Dose Cohorts and Food Conditions

|  | Single Ascending Dose Cohorts | | | | | | | | | | Food Effect Study | | | |
| --- | --- | --- | --- | --- | --- | --- | --- | --- | --- | --- | --- | --- | --- | --- |
| VU319 Dose | **60 mg** | | **120 mg** | | **240 mg** | | **400 mg** | | **600 mg** | | **120 mg Fasted Condition** | | **120 mg Fed Condition** | |
| PK Parameter | **Mean (SD)** | **N** | **Mean (SD)** | **N** | **Mean (SD)** | **N** | **Mean (SD)** | **N** | **Mean (SD)** | **N** | **Mean (SD)** | **N** | **Mean (SD)** | **N** |
| Max_Rate (mg/h) | 0.0024 (0.0012) | 3 | 0.0032 (0.0025) | 6 | 0.0053 (0.0024) | 6 | 0.0056 (0.0018) | 6 | 0.0051 (0.0027) | 6 | 0.0025 (0.0011) | 10 | 0.005 (0.003) | 10 |
| T_max_Rate_ (h)^a^ | 3.5 (3.5, 8.5) | 3 | 8.5 (3.5, 18.0) | 6 | 6.0 (3.5, 18.0) | 6 | 6.0 (3.5, 30.0) | 6 | 6.0 (3.5, 42.0) | 6 | 8.5 (3.5, 30) | 10 | 6.0 (1.0, 8.5) | 10 |
| Total Ae (mg) | 0.045 (0.021) | 3 | 0.061 (0.032) | 6 | 0.093 (0.042) | 6 | 0.12 (0.058) | 6 | 0.143 (0.065) | 6 | 0.068 (0.026) | 10 | 0.091 (0.034) | 10 |
| Total %fe (%) | 0.075 (0.034) | 3 | 0.051 (0.026) | 6 | 0.039 (0.017) | 6 | 0.0297 (0.015) | 6 | 0.024 (0.011) | 6 | 0.056 (0.22) | 10 | 0.075 (0.028) | 10 |
| CL_R_ (mL/h) | 1.25 (0.303) | 3 | 1.16 (0.537) | 5 | 1.13 (0.657) | 4 | 0.97 (0.42) | 4 | 0.673 (0.34) | 6 | 1.24 (0.36) | 8 | 1.48 (0.4) | 9 |
| CL_NR_ (mL/h) | 1887 (850) | 3 | 2067 (275) | 5 | 3148 (581) | 4 | 3419 (961) | 4 | 2866 (765) | 6 | 2347 (1150) | 8 | 2004 (846) | 9 |
| CL_ratio_ | 0.00075 (0.00034) | 3 | 0.00057 (0.00025) | 5 | 0.00034 (0.00019) | 4 | 0.00031 (0.00019) | 4 | 0.00024 (0.00011) | 6 | 0.00059 (0.00023) | 8 | 0.0008 (0.00025) | 9 |

Notes. Abbreviations: CL_R_ = renal clearance of drug; CL_NR_ = Nonrenal clearance of drug; CL_ratio_ = ratio of renal clearance to apparent systemic clearance; Max_Rate = maximum observed excretion rate; T_max_Rate_ = time to maximum observed excretion rate; Total %fe = percentage of cumulative fraction of the dose excreted unchanged in urine over the entire collection interval; Total Ae=cumulative amount of drug/analyte excreted in urine over the entire collection interval; N = number of subjects; PK = pharmacokinetic; SD = standard deviation. ^a^T_max_Rate_ values are presented as median (minimum, maximum).

Supplementary Table 3. Urine Pharmacokinetic Parameters of VU424 Across Single Ascending Dose Cohorts and Food Conditions

|  | Single Ascending Dose Cohorts | | | | | | | | | | Food Effect Study | | | |
| --- | --- | --- | --- | --- | --- | --- | --- | --- | --- | --- | --- | --- | --- | --- |
| VU319 Dose | **60 mg** | | **120 mg** | | **240 mg** | | **400 mg** | | **600 mg** | | **120 mg Fasted Condition** | | **120 mg Fed Condition** | |
| PK Parameter | **Mean (SD)** | **N** | **Mean (SD)** | **N** | **Mean (SD)** | **N** | **Mean (SD)** | **N** | **Mean (SD)** | **N** | **Mean (SD)** | **N** | **Mean (SD)** | **N** |
| Max_Rate (mg/h) | 0.008 (0.0042) | 3 | 0.011 (0.014) | 6 | 0.015 (0.0069) | 6 | 0.043 (0.037) | 6 | 0.065 (0.047) | 6 | 0.0091 (0.0033) | 10 | 0.013 (0.0072) | 10 |
| T_max_Rate_ (h)^a^ | 30.0 (8.5, 30.0) | 3 | 24.0 (8.5, 30.0) | 6 | 8.5 (3.5, 30.0) | 6 | 30.0 (3.5, 30.0) | 6 | 30.0 (8.5, 42.0) | 6 | 30.0 (8.5, 42.0) | 10 | 30.0 (3.5, 42.0) | 10 |
| Total Ae (mg) | 0.17 (0.09) | 3 | 0.204 (0.2) | 6 | 0.33 (0.18) | 6 | 0.93 (0.81) | 6 | 1.88 (1.53) | 6 | 0.27 (0.12) | 10 | 0.33 (0.16) | 10 |

Notes. Abbreviations: CL_R_ = renal clearance of drug; CL_NR_ = Nonrenal clearance of drug; CL_ratio_ = ratio of renal clearance to apparent systemic clearance; Max_Rate = maximum observed excretion rate; T_max_Rate_ = time to maximum observed excretion rate; Total %fe = percentage of cumulative fraction of the dose excreted unchanged in urine over the entire collection interval; Total Ae=cumulative amount of drug/analyte excreted in urine over the entire collection interval; N = number of subjects; PK = pharmacokinetic; SD = standard deviation. ^a^T_max_Rate_ values are presented as median (minimum, maximum).

Supplementary Table 4. Treatment-Emergent Adverse Events by System Organ Class and Preferred Term Single Ascending Dose Study and Food Effect Study – Safety Analysis Set

|  | Single Ascending Dose Study | | | | | | | | | | | | | Food Effect Study | | | | | | |
| --- | --- | --- | --- | --- | --- | --- | --- | --- | --- | --- | --- | --- | --- | --- | --- | --- | --- | --- | --- | --- |
| MedDRA System Organ Class  Preferred Term | **Placebo SAD (N=10)** | | **VU319 60 mg (N=6)** | | **VU319 120 mg (N=6)** | | **VU319 240 mg (N=6)** | | **VU319 400 mg (N=6)** | | **VU319 600 mg (N=6)** | | | **Placebo Food Effect (N=4)** | | **VU319 120mg Fasted (N=10)** | | **VU319 120mg Fed**  **(N=10)** | |  |
|  | **Participants n (%)** | **Events n** | **Participants n (%)** | **Events n** | **Participants n (%)** | **Events n** | **Participants n (%)** | **Events n** | **Participants n (%)** | **Events n** | **Participants n (%)** | **Events n** | **Participants n (%)** | | **Events**  **n** | **Participants n (%)** | **Events**  **n** | **Participants n (%)** | **Events**  **n** |  |
| Subjects with any TEAE | 6 (60.0) | 14 | 4 (66.7) | 9 | 3 (50.0) | 5 | 4 (66.7) | 5 | 4 (66.7) | 6 | 4 (66.7) | 8 | 3 (75.0) | | 6 | 7 (70.0) | 10 | 2 (20.0) | 4 |  |
| Ear and labyrinth disorders | 1 (10.0) | 1 | - - | - - | - - | - - | - - | - - | - - | - - | - - | - - | - - | | - - | - - | - - | - - | - - |  |
| Tinnitus | 1 (10.0) | 1 | - - | - - | - - | - - | - - | - - | - - | - - | - - | - - | - - | | - - | - - | - - | - - | - - |  |
| Eye disorders | - - | - - | - - | - - | - - | - - | 1 (16.7) | 1 | - - | - - | 1 (16.7) | 1 | - - | | - - | - - | - - | 1 (10.0) | 1 |  |
| Dry eye | - - | - - | - - | - - | - - | - - | - - | - - | - - | - - | 1 (16.7) | 1 | - - | | - - | - - | - - | - - | - - |  |
| Vision blurred | - - | - - | - - | - - | - - | - - | 1 (16.7) | 1 | - - | - - | - - | - - | - - | | - - | - - | - - | - - | - - |  |
| Gastrointestinal disorders | 3 (30.0) | 5 | 1 (16.7) | 1 | 1 (16.7) | 1 | - - | - - | - - | - - | - - | - - | - - | | - - | 1 (10.0) | 1 | - - | - - |  |
| Nausea | 2 (20.0) | 2 | 1 (16.7) | 1 | 1 (16.7) | 1 | - - | - - | - - | - - | - - | - - | - - | | - - | - - | - - | - - | - - |  |
| Constipation | 1 (10.0) | 1 | - - | - - | - - | - - | - - | - - | - - | - - | - - | - - | - - | | - - | - - | - - | - - | - - |  |
| Diarrhea | 1 (10.0) | 1 | - - | - - | - - | - - | - - | - - | - - | - - | - - | - - | - - | | - - | - - | - - | - - | - - |  |
| Dyspepsia | 1 (10.0) | 1 | - - | - - | - - | - - | - - | - - | - - | - - | - - | - - | - - | | - - | - - | - - | - - | - - |  |
| General disorders and administration site conditions | 1 (10.0) | 1 | - - | - - | 1 (16.7) | 1 | - - | - - | - - | - - | - - | - - | - - | | - - | - - | - - | - - | - - |  |
| Feeling cold | - - | - - | - - | - - | 1 (16.7) | 1 | - - | - - | - - | - - | - - | - - | - - | | - - | - - | - - | - - | - - |  |
| Feeling hot | 1 (10.0) | 1 | - - | - - | - - | - - | - - | - - | - - | - - | - - | - - | - - | | - - | - - | - - | - - | - - |  |
| Abdominal Discomfort | - - | - - | - - | - - | - - | - - | - - | - - | - - | - - | - - | - - | - - | | - - | 1 (10.0) | 1 | - - | - - |  |
| Investigations | - - | - - | 1 (16.7) | 1 | - - | - - | - - | - - | - - | - - | - - | - - | - - | | - - | 1 (10.0) | 1 | 1 (10.0) | 2 |  |
| Blood bilirubin increased | - - | - - | 1 (16.7) | 1 | - - | - - | - - | - - | - - | - - | - - | - - | - - | | - - | - - | - - | - - | - - |  |
| Alanine aminotransferase Increased | - - | - - | - - | - - | - - | - - | - - | - - | - - | - - | - - | - - | - - | | - - | - - | - - | 1 (10.0) | 1 |  |
| Aspartate aminotransferase Increased | - - | - - | - - | - - | - - | - - | - - | - - | - - | - - | - - | - - | - - | | - - | - - | - - | 1 (10.0) | 1 |  |
| Urine Analysis Abnormal | - - | - - | - - | - - | - - | - - | - - | - - | - - | - - | - - | - - | - - | | - - | 1 (10.0) | 1 | - - | - - |  |
| Musculoskeletal and connective tissue disorders | 1 (10.0) | 1 | - - | - - | - - | - - | - - | - - | 1 (16.7) | 1 | - - | - - | 1 (25.0) | | 1 | 1 (10.0) | 2 | - - | - - |  |
| Musculoskeletal stiffness | 1 (10.0) | 1 | - - | - - | - - | - - | - - | - - | - - | - - | - - | - - | - - | | - - | - - | - - | - - | - - |  |
| Myalgia | - - | - - | - - | - - | - - | - - | - - | - - | 1 (16.7) | 1 | - - | - - | - - | | - - | 1 (10) | 2 | - - | - - |  |
| Arthralgia | - - | - - | - - | - - | - - | - - | - - | - - | - - | - - | - - | - - | 1 (25.0) | | 1 | - - | - - | - - | - - |  |
| Nervous system disorders | 2 (20.0) | 4 | 3 (50.0) | 6 | 3 (50.0) | 3 | 3 (50.0) | 4 | 3 (50.0) | 4 | 4 (66.7) | 7 | 3 (75.0) | | 5 | 4 (40.0) | 5 | 1 (10.0) | 1 |  |
| Headache | 2 (20.0) | 4 | 3 (50.0) | 6 | 2 (33.3) | 2 | 1 (16.7) | 1 | 3 (50.0) | 4 | 3 (50.0) | 6 | 3 (75.0) | | 5 | 4 (40.0) | 5 | 1 (10.0) | 1 |  |
| Dizziness | - - | - - | - - | - - | - - | - - | 3 (50.0) | 3 | - - | - - | - - | - - | - - | | - - | - - | - - | - - | - - |  |
| Dysgeusia | - - | - - | - - | - - | - - | - - | - - | - - | - - | - - | 1 (16.7) | 1 | - - | | - - | - - | - - | - - | - - |  |
| Hypoaesthesia | - - | - - | - - | - - | 1 (16.7) | 1 | - - | - - | - - | - - | - - | - - | - - | | - - | - - | - - | - - | - - |  |
| Psychiatric disorders | - - | - - | - - | - - | - - | - - | - - | - - | 1 (16.7) | 1 | - - | - - | - - | | - - | - - | - - | - - | - - |  |
| Hallucination, olfactory | - - | - - | - - | - - | - - | - - | - - | - - | 1 (16.7) | 1 | - - | - - | - - | | - - | - - | - - | - - | - - |  |
| Respiratory, thoracic and mediastinal disorders | 2 (20.0) | 2 | - - | - - | - - | - - | - - | - - | - - | - - | - - | - - | - - | | - - | - - | - - | - - | - - |  |
| Rhinorrhoea | 2 (20.0) | 2 | - - | - - | - - | - - | - - | - - | - - | - - | - - | - - | - - | | - - | - - | - - | - - | - - |  |
| Skin and subcutaneous tissue disorders | - - | - - | 1 (16.7) | 1 | - - | - - | - - | - - | - - | - - | - - | - - | - - | | - - | 1 (10.0) | 1 | - - | - - |  |
| Hyperhidrosis | - - | - - | 1 (16.7) | 1 | - - | - - | - - | - - | - - | - - | - - | - - | - - | | - - | - - | - - | - - | - - |  |
| Rash | - - | - - | - - | - - | - - | - - | - - | - - | - - | - - | - - | - - | - - | | - - | 1 (10.0) | - - | - - | - - |  |

Abbreviations: MedDRA = Medical Dictionary for Regulatory Activities; N = number of participants in each group; n = number of participants meeting the criterion; TEAE = treatment-emergent adverse event. Notes: Each category of disorder is listed first at the beginning of each subsection of the table with all individual disorders listed after. The number of participants (N) for the given dose group is used as the denominator for calculating percentages for the participant counts. A TEAE is an adverse event with onset date on or after the first study dose date and no later than last study dose date + 30 days. A participant may have more than one TEAE per category. A participant is counted at most once per category. For events, all occurrences of TEAEs are counted per category.

Supplementary Table 5. ECG Datapoints Across the Single Ascending Dose and Food Effect Studies

|  | Single Ascending Dose Study Cohorts | | | | | | Food Effect Study: 120 mg | | | |
| --- | --- | --- | --- | --- | --- | --- | --- | --- | --- | --- |
| ECG Parameter and Timepoint | **Placebo  (N=10)** | **60 mg (N=6)** | **120 mg (N=6)** | **240 mg (N=6)** | **400 mg (N=6)** | **600 mg (N=6)** | **Placebo Fasted or Fed (N=4)** | **Fasted (N=10)** | **Fed (N=10)** |  |
| QTcF |  |  |  |  |  |  |  |  |  |  |
| Baseline | 409.2 (12.59) | 418.0 (17.1) | 406.0 (12.23) | 426.0 (33.22) | 401.8 (24.13) | 416.0 (25.35) | 421.0 (10.46) | 408.9 (20.1) | 406.7 (21.7) |  |
| +1 hour | 412.6 (16.75) | 411.0 (11.82) | 409.7 (15.34) | 422.7 (22.05) | 405.3 (24.45) | -- | -- | -- | -- |  |
| +2 hour | 406.5 (17.72) | 414.8 (18.13) | 407.0 (21.02) | 415.5 (28.46) | 405.0 (26.62) | 419.7 (27.65) | 423.3 (5.12) | 405.0 (22.7) | 399.2 (19.09) |  |
| +3 hour | 409.2 (17.86) | 412.8 (23.01) | 412.7 (20.02) | 421.7 (19.48) | 405.3 (25.65) | 419.5 (31.48) | 422.3 (8.66) | 405.9 (19.13) | 402.3 (24.08) |  |
| +5 hour | 403.4 (17.97) | 417.2 (19.47) | 406.8 (15.5) | 414.8 (24.28) | 405.2 (27.72) | 413.8 (23.37) | 427.0 (11.02) | 406.4 (18.05) | 405.0 (16.01) |  |
| +9 hour | 401.8 (13.56) | 411.3 (21.31) | 409.2 (16.68) | 421.0 (15.03) | 400.7 (23.41) | 413.8 (26.54) | 422.0 (12.3) | 405.5 (14.4) | 402.5 (19.51) |  |
| +24 hour | 399.3 (14.87) | 408.7 (8.52) | 408.3 (12.64) | 415.2 (16.22) | 400.7 (17.13) | 408.0 (17.38) | 415.5 (7.51) | 401.2 (19.21) | 403.0 (16.89) |  |
| +36 hour | 403.4 (12.29) | 410.6 (10.5)* | 408.0 (18.17) | 418.2 (24.29) | 404.5 (20.16) | 418.2 (24.79) | 419.0 (6.68) | 405.2 (23.74) | 399.5 (21.91) |  |
| +72 hour | 392.5 (17.68)^ | -- | -- | -- | -- | 413.3 (19.22) | 412.8 (17.33) | 403.2 (16.39) | 402.7 (17.38)^#^ |  |
| +144 hour | 412.5 (17.12) | 415.5 (7.66) | 406.7 (17.95) | 422.8 (12.32) | 410.5 (10.21) | 415.7 (23.99) | 414.5 (16.82) | 405.1 (19.72) | 403.6 (24.86) |  |
|  |  |  |  |  |  |  |  |  |  |  |
|  |  |  |  |  |  |  |  |  |  |  |

Notes: Data is presented as mean (standard deviation). QTcF = QT interval corrected using Fridericia’s method. *n = 5; ^n = 2; ^#^n = 9.

Supplementary Table 6. Main Cognitive Outcome Measures Across Dosage Groups

|  |  | VU319 Dose | | | | | | | | | | | |
| --- | --- | --- | --- | --- | --- | --- | --- | --- | --- | --- | --- | --- | --- |
| Cognitive Task | **Outcome Measure** | **Placebo**  **Pre-Drug** | **Post-Drug** | **60 mg**  **Pre-Drug** | **Post-Drug** | **120 mg**  **Pre-Drug** | **Post-Drug** | **240 mg**  **Pre-Drug** | **Post-Drug** | **400 mg**  **Pre-Drug** | **Post-Drug** | **600 mg**  **Pre-Drug** | **Post Drug** |
| CFF | Median Ascending score (Hz) | 35.88 (2.1) | 37.13 (2.1) | 36.32 (4.6) | 37.67 (3.1) | 37.33 (2.38) | 36.53 (3.87) | 32.72 (9.97) | 38.12 (4.1) | 37.92 (3.7) | 39.98 (3.97) | 35.45 (3.61) | 36.58 (3.14) |
|  | Median Descending score | 39.31 (5.23) | 38.73 (3.52) | 37 (5.35) | 38.32 (7.51) | 40.23 (4.95) | 38.68 (3.31) | 37.1 (6.52) | 37.25 (6.89) | 39.67 (2.1) | 40.05 (3.01) | 39.22 (5.92) | 39.73 (5.18) |
| CRT | Median Total Time (ms) | 746.75 (101.8) | 703.4 (76.8) | 735.08 (60.61) | 734.25 (120.68) | 718.58 (103.36) | 735.0.8 (136.58) | 800.92 (121.25) | 759.42 (151.55) | 731 (55.14) | 728.92 (57.1) | 662.17 (61.66) | 649.58 (71.76) |
|  | Median Recognition Time | 398.3 (48.43) | 382.45 (35.85) | 391.33 (59.93) | 394.92 (82.56) | 384.92 (42.09) | 389 (43.6) | 410.5 (68.61) | 399 (83.54) | 396.67 (41.84) | 388.17 (40.47) | 364.92 (18.0) | 362.67 (52.01) |
|  | Median Motor Time | 331.8 (64.25) | 308.5 (54.16) | 331.08 (35.75) | 330.42 (64.58) | 329.08 (80.22) | 332.67 (89.6) | 374.58 (64.5) | 353.25 (69.11) | 331 (38.55) | 334.33 (33) | 291.42 (46.98) | 282.33 (56.95) |
| CPT | Hit Response Time (ms) | 383.75 (53.23) | 380.66 (47.07) | 393.27 (46.65) | 407.08 (66.44) | 361.23 (27.25) | 356.2 (29.58) | 372.21 (43.06) | 372.94 (64.52) | 374.41 (25.8) | 365.85 (33.44) | 387.39 (51.74) | 356.18 (45.08) |
|  | Omission Errors | 2 (2.98) | 2.9 (2.08) | 1 (1.27) | 3.33 (4.55) | 0.83 (0.75) | 4.67 (5.28) | 1 (1.27) | 2.33 (2.58) | 0.67 (1.2) | 1.67 (1.97) | 1.33 (3.27) | 1.5 (2.35) |
|  | Commission Errors | 14 (5.7) | 12 (6.09) | 12.83 (4.07) | 13.33 (4.37) | 15.5 (6.66) | 15.33 (5.92) | 11.33 (3.62) | 12 (1.55) | 10.33 (2.81) | 9.5 (3.08) | 13 (5.66) | 17 (8.49) |
|  | Perseverations | 0.3 (0.68) | 0.2 (0.42) | 0.33 (0.52) | 0 (0) | 0 (0) | 1.5 (1.98) | 0.5 (1.23) | 0.83 (1.6) | 0.17 (0.41) | 0.33 (0.82) | 0.33 (0.82) | 1.5 (3.21) |
| SSAT | Alerting Cost (ms) | 39.25 (15.69) | 23.25 (40.71) | 53.67 (39.48) | 59.58 (43) | 62.75 (45) | 44.25 (22.86) | 34.83 (27.3) | 58.83 (38.04) | 59.75 (24.51) | 44.92 (26.61) | 26.08 (37.44) | 19.5 (41.26) |
|  | Reorienting Cost | 26.95 (33.86) | 38 (40.93) | 43.58 (31.92) | 55.25 (34.49) | 10.17 (18.88) | 20.33 (32.95) | 27.92 (29.28) | 39.67 (33.04) | 22.17 (25) | 31.25 (18.61) | 20.92 (11.57) | 31.33 (15.75) |
| N-Back | 0-back Sensitivity (*d’*) | 5.63 (0.73) | 5.58 (0.7) | 5.64 (0.84) | 5.37 (1.26) | 5.64 (0.84) | 5.37 (1.26) | 6.18 (0) | 6.18 (0) | 6.18 (0) | 6.18 (0) | 5.57 (0.95) | 5.27 (1.43) |
|  | 1-back Sensitivity | 5.21 (0.89) | 4.98 (1.33) | 5.01 (1.17) | 4.99 (1.48) | 4.91 (1.08) | 4.46 (1.35) | 4.72 (2.02) | 4.32 (2.3) | 5.64 (0.84) | 5.37 (1.26) | 5.07 (0.59) | 5.09 (1.06) |
|  | 2-back Sensitivity | 4.06 (1.59) | 3.83 (1.93) | 3.35 (0.91) | 3.12 (1.22) | 3.28 (1.98) | 3.25 (2.19) | 3.77 (1.84) | 3.42 (2.29) | 3.54 (1.69) | 3.6 (1.84) | 4.31 (1.69) | 4.43 (1.72) |
|  | 3-back Sensitivity | 3.07 (1.35) | 2.93 (1.88) | 2.65 (0.49) | 2.11 (0.36) | 2.34 (1.12) | 1.75 (1.14) | 2.37 (0.81) | 1.66 (0.83) | 2.34 (0.74) | 2.01 (0.74) | 3.3 (1.02) | 2.94 (1.3) |
| SRT | Immediate Recall | 88 (11.59) | 76.6 (15.06) | 79.83 (13.18) | 71.67 (14.95) | 84.67 (20.24) | 84 (19.55) | 87.33 (11.38) | 80.67 (13.65) | 76.33 (24.51) | 70.83 (16.03) | 91.17 (11.81) | 85.83 (21.7) |
|  | Recall Failure | 9.2 (5.83) | 17.2 (10.47) | 13.5 (4.46) | 19.83 (9.37) | 13 (9.76) | 12.5 (10.78) | 10.83 (7.73) | 10.83 (10.83) | 19 (15.66) | 20 (11.15) | 8.17 (5.27) | 10.67 (12.58) |
|  | Recall Consistency | 53.4 (15.57) | 41.2 (18.57) | 44 (22.93) | 35.33 (18.32) | 51.67 (27.6) | 49.33 (25.15) | 54.33 (13.31) | 41.5 (16.11) | 42.33 (29.85) | 33.33 (18.24) | 59.83 (13.39) | 51 (27.8) |
|  | Delayed Recall | 10.8 (2.7) | 8.2 (4.76) | 9 (3.52) | 8.83 (3.55) | 10.17 (5.42) | 9.5 (5.86) | 10.67 (1.86) | 9 (2.45) | 8.83 (4.79) | 7.83 (3.87) | 11.83 (1.94) | 8.67 (4.8) |

Notes: Data is presented as mean (standard deviation). CFF, critical flicker fusion task, CRT, choice reaction time task, CPT, Conners’ continuous performance task, SSAT, spatial selective attention task, SRT, selective reminding task.

Supplementary Table 7. Main Cognitive Outcome Measures Across Fed and Fasting Conditions

| Cognitive Task | Outcome Measure | Fasting  Pre-Drug | Post-Drug | Fed  Pre-Drug | Post-Drug |
| --- | --- | --- | --- | --- | --- |
| CFF | Median Ascending score (Hz) | 34.62 (9.06) | 39.03 (5.2) | 39.09 (5.81) | 39.29 (4.38) |
|  | Median Descending score | 38.49 (5.19) | 38.59 (4.57) | 39.25 (3.25) | 39.36 (4.08) |
| CRT | Median Total Time (ms) | 691.25 (64.17) | 679.55 (35.68) | 695.7 (47.32) | 673.65 (47.86) |
|  | Median Recognition Time | 384.9 (53.58) | 370.55 (33.17) | 365.45 (41.27) | 369.4 (41.57) |
|  | Median Motor Time | 302 (40.02) | 299.2 (24.19) | 321.5 (36.67) | 297.25 (36.2) |
| CPT* | Hit Response Time (ms) | 395.78 (51.66) | 387.85 (64.33) | 381.6 (59.56) | 381.1 (71.8) |
|  | Omission Errors | 4.56 (10.4) | 4.33 (7.16) | 1.22 (1.48) | 2 (4.24) |
|  | Commission Errors | 11.6 (5.68) | 12.6 (6.62) | 11.1 (5.58) | 12.3 (6.2) |
|  | Perseverations | 3.89 (11.7) | 2.56 (6.93) | 0.44 (1.01) | 1.78 (2.44) |
| SSAT | Alerting Cost (ms) | 60.4 (53.4) | 25.8 (59.97) | 54.1 (45.3) | 113.15 (181.48) |
|  | Reorienting Cost | 52.15 (24.83) | 49.55 (35.68) | 56.75 (38.19) | 49.45 (32.65) |
| N-Back | 0-back Sensitivity (*d’*) | 4.64 (1.22) | 5.15 (1.1) | 4.67 (1.4) | 4.62 (1.32) |
|  | 1-back Sensitivity | 5.01 (0.93) | 3.38 (2.09) | 5.42 (0.99) | 4.13 (1.6) |
|  | 2-back Sensitivity | 3.79 (1.18) | 2.3 (1.64) | 3.75 (1.21) | 2.89 (1.42) |
|  | 3-back Sensitivity | 2.55 (1.42) | 1.96 (1.4) | 2.29 (0.65) | 2.53 (0.97) |
| SRT | Immediate Recall | 86.6 (17.61) | 88.9 (19.83) | 88.7 (13.59) | 88.9 (20.45) |
|  | Recall Failure | 9.4 (8.54) | 10.2 (9.74) | 8 (6.11) | 8.3 (10.58) |
|  | Recall Consistency | 51.1 (24.64) | 55.7 (26.22) | 54 (18.33) | 53.4 (27.22) |
|  | Delayed Recall | 11.5 (3.69) | 11.3 (3.97) | 11.2 (3.33) | 10.9 (3.92) |

Notes: Data is presented as mean (standard deviation). CFF, critical flicker fusion task, CRT, choice reaction time task, CPT, Conners’ continuous performance task, SSAT, spatial selective attention task, SRT, selective reminding task. *n = 9.

Supplementary Table 8. Mean Amplitudes Over the Parietal Cortex for all Trial Types, Time Points and Dosage Groups

|  |  |  |  |  |  |  |  |  |  |  |  |  |  |
| --- | --- | --- | --- | --- | --- | --- | --- | --- | --- | --- | --- | --- | --- |
| Cognitive Task | **Trial Type** | **Placebo**  **Pre-Drug** | **Post-Drug** | **60mg**  **Pre-Drug** | **Post-Drug** | **120mg**  **Pre-Drug** | **Post-Drug** | **240mg**  **Pre-Drug** | **Post-Drug** | **400mg**  **Pre-Drug** | **Post-Drug** | **600mg**  **Pre-Drug** | **Post-Drug** |
| Auditory Oddball | Standard | 0.46 (1.65) | 1.03 (1.71) | 1.23 (0.96) | 1.31 (1.02) | 1.83 (1.04) | 0.85 (1.41) | 1.34 (1.02) | 1.04 (1.54) | 0.66 (1.09) | 0.48 (0.92) | 0.74 (1.24) | 1.84 (1.22) |
|  | Target | 2.2 (1.43) | 2.44 (1.86) | 2.0 (1.32) | 3.69 (1.83) | 3.23 (1.85) | 3.18 (1.24) | 2.94 (1.57) | 2.42 (1.53) | 1.7 (0.67) | 2.12 (0.34) | 3.08 (2.61) | 4.31 (2.13) |
| Visual Oddball | Standard | 0.31 (1.48)* | 0.48 (1.59) | -0.13 (1.23) | -0.66 (1.57) | 0.99 (1.12)^#^ | 0.01 (1.05)^#^ | 0.32 (1.04) | 1.02 (1.44)^#^ | 1.04 (1.03) | 1.48 (0.73) | 1.46 (2.51) | 1.47 (1.1) |
|  | Target | 1.44 (1.49)* | 2.04 (1.22) | 1.19 (1.9) | 1.36 (0.91) | 2.71 (1.58)^#^ | 1.5 (1.26)^#^ | 1.93 (1.88) | 2.2 (2.28)^#^ | 1.75 (1.5) | 2.7 (1.92) | 3.46 (2.31) | 3.1 (2.23) |
| Incidental Memory task | Novel | 1.64 (1.58) | 1.22 (2.17) | 0.49 (2.16) | 0.87 (1.92) | 2.3 (1.78)^#^ | 1.67 (2.01) | 1.22 (1.44) | 1.76 (1.4) | 2.35 (1.07) | 2.45 (1.79) | 2.47 (1.3) | 1.98 (0.97) |
|  | Repeat | 1.63 (1.89) | 1.13 (2.31) | 0.77 (1.85) | 1.25 (1.02) | 2.66 (2.5)^#^ | 2.16 (1.69) | 2.14 (1.27) | 2.19 (1.37) | 1.91 (1.19) | 3.14 (2.3) | 2.74 (1.63) | 3.17 (1.37) |

Notes: Mean (Standard Deviation). Amplitudes are in µV. *n = 9; ^#^n = 5.

Supplementary Table 9. Mean Amplitudes Over the Parietal Cortex for all Trial Types, Time Points and Food Conditions.

| Task | Trial Type | Fasting  Pre-Drug | Post-Drug | Fed  Pre-Drug | Post-Drug |
| --- | --- | --- | --- | --- | --- |
| Auditory Oddball* | Standard | 1.09 (1.78) | 1.17 (1.66) | 0.51 (1.65) | -0.07 (0.96) |
|  | Target | 2.11 (2.11) | 2.56 (2.38) | 2.32 (1.46) | 0.8 (1.11) |
| Visual Oddball* | Standard | -0.16 (1.28) | -0.16 (1.73) | -0.29 (1.17) | 0.25 (0.62) |
|  | Target | 0.71 (1.6) | 0.72 (3.1) | 0.91 (1.49) | 1.2 (.29) |
| Incidental Memory Task | Novel | 1.34 (1.09) | 1.0 (1.39) | 1.0 (0.95) | 1.04 (1.81) |
|  | Repeat | 1.46 (1.45) | 1.48 (1.3) | 0.88 (2.27) | 2.43 (1.59) |

Notes: Mean (Standard Deviation). Amplitudes are in µV. *n = 9.
